# 1-(3-Fluoro­phen­yl)-3-(4-nitro­phen­yl)urea

**DOI:** 10.1107/S160053681202507X

**Published:** 2012-06-13

**Authors:** Mu-Sen Lin, Yu Shi, Shi-Yu Zhang, Yi-Liang Li

**Affiliations:** a School of Chinese Materia Medica, Tianjin University of Traditional Chinese Medicine, Tianjin, 300193, People’s Republic of China; b Tianjin Key Laboratory of Molecular Design and Drug Discovery, Tianjin Institute of Pharmaceutical Research, Tianjin, 300193, People’s Republic of China

## Abstract

In the title compound, C_13_H_10_FN_3_O_3_, the dihedral angle between the fluoro­phenyl and nitro­phenyl ring planes is 6.51 (9)°. The crystal structure features N—H⋯O hydrogen bonds.

## Related literature

The title compound is an activated fragment of sorafenib derivatives. Sorafenib is a VEGFR-2 inhibitor (Ferrara *et al.*, 2003 ▶; Peruzzi *et al.*, 2006 ▶) that has good therapeutic effect for renal carcinoma and liver cancer (Wan *et al.*, 2004 ▶; Wilhelm *et al.*, 2004 ▶).
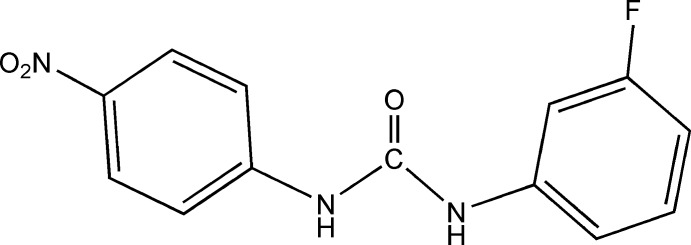


## Experimental

### 

#### Crystal data

C_13_H_10_FN_3_O_3_
*M* *_r_* = 275.24
Monoclinic, 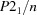

*a* = 8.351 (4) Å
*b* = 12.461 (6) Å
*c* = 11.912 (6) Å
β = 100.315 (9)°
*V* = 1219.5 (11) Å^3^
*Z* = 4
Mo *K*α radiation
μ = 0.12 mm^−1^
*T* = 113 K
0.24 × 0.22 × 0.20 mm

#### Data collection

Rigaku Saturn CCD area-detector diffractometer
Absorption correction: multi-scan *CrystalClear* *T*_min_ = 0.972, *T*_max_ = 0.977
12466 measured reflections
2900 independent reflections
2459 reflections with *I* > 2σ(*I*)
*R*_int_ = 0.045

#### Refinement

*R*[*F* ^2^ > 2σ(*F* ^2^)] = 0.052
*wR*(*F* ^2^) = 0.131
*S* = 1.12
2900 reflections
189 parameters
2 restraints
H atoms treated by a mixture of independent and constrained refinement
Δρ_max_ = 0.30 e Å^−3^
Δρ_min_ = −0.26 e Å^−3^

### 

Data collection: *CrystalClear* (Rigaku, 2005 ▶); cell refinement: *CrystalClear*; data reduction: *CrystalClear*; program(s) used to solve structure: *SHELXS97* (Sheldrick, 2008 ▶); program(s) used to refine structure: *SHELXL97* (Sheldrick, 2008 ▶); molecular graphics: *SHELXTL/PC* (Sheldrick, 2008 ▶); software used to prepare material for publication: *SHELXTL/PC*.

## Supplementary Material

Crystal structure: contains datablock(s) I, global. DOI: 10.1107/S160053681202507X/zj2072sup1.cif

Structure factors: contains datablock(s) I. DOI: 10.1107/S160053681202507X/zj2072Isup2.hkl

Supplementary material file. DOI: 10.1107/S160053681202507X/zj2072Isup3.cml

Additional supplementary materials:  crystallographic information; 3D view; checkCIF report

## Figures and Tables

**Table 1:** Hydrogen-bond geometry (Å, °)

*D*—H⋯*A*	*D*—H	H⋯*A*	*D*⋯*A*	*D*—H⋯*A*
N2—H2*A*⋯O1^i^	0.91 (1)	1.99 (1)	2.890 (2)	170 (2)
N3—H3*A*⋯O2^i^	0.90 (1)	2.28 (1)	3.157 (2)	168 (2)

Symmetry code: (i) 
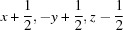
.

**Fig. 1.**
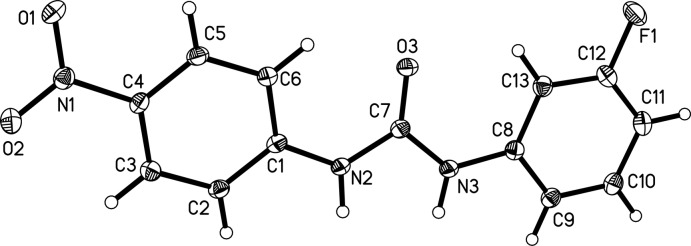
The molecular structure of the title compound, showing the atomic numbering scheme. Displacement ellipsoids are drawn at the 30% probability level.

**Fig. 2.**
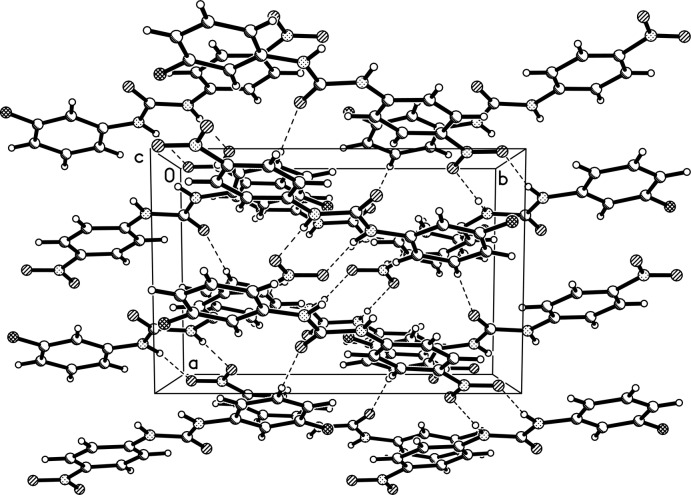
Packing diagram of the title compound viewed along the c axis. Hydrogen bonds are shown as dashed lines.

## supplementary crystallographic information

### Comment

Sorafenib is a VEGFR-2 inhibitor (Ferrara *et al.*, 2003; Peruzzi *et al.*, 2006) that has good therapeutic effect for renal carcinoma and liver cancer (Wan *et al.*, 2004; Wilhelm *et al.*, 2004). 1-(3-fluorophenyl)-3-(4-nitrophenyl) urea is an important activated fragment of sorafenib derivatives. We present here the structure characterization of the title compound.

In the molecule of the title compound (Fig.1) bond lengths and angles have normal values. The interplanar angle between the fluorobenzyl and nitrobenzyl ring planes is 6.51 (9)°. The crystal structure is stabilized by the intermolecular N—H···O hydrogen bonds. The crystal structure (Fig.2) is stabilized by intermolecular N—H···O hydrogen bonds (table 1).

### Experimental

A solution of 4-nitroaniline (1.38 g, 10 mmol) in DCM (100 ml) was added dropwise to a stirred solution of bis(trichloromethyl) carbonate (5.92 g, 20 mmol) in DCM (20 ml) at the atmosphere of ice-bath.The reaction mixture was stirred for 2 hrs at 0–5°C. Then the reaction mixture was added drpwise to a refluxed and stirred solution of 3-fluoroaniline (1.11 g, 10 mmol) in DCM (40 ml).The reaction was completed within 2 hrs at the reflux temperature.The solvent was removed under reduced pressure.Acetone (100 ml) and H_2_O (300 ml) was added to the mixture. The solid was collected and washed with H_2_O,then gave a yellow solid. The yield was 2.08 g (75.6%). Put about 0.3 g of the product in the ampoule bottle and add 10 ml absolute ethyl alcohol, yellow single crystals suitable for X-ray diffraction analysis were obtained by slow evaporation of the solvent at room temperature after 3 weeks.

### Refinement

All H atoms were detected in a difference map, nevertheless, the H-atoms attached to the nitrogen atoms were refined freely, and the H-atoms attached to the carbon atoms were placed in calculated positions and refined using a riding motion approximation, with C—H=0.95 Å, with *U*_iso_(H)=1.2*U*_eq_(C).

### Crystal data

**Table:**

C_13_H_10_FN_3_O_3_	*F*(000) = 568
*M**_r_* = 275.24	*D*_x_ = 1.499 Mg m^−^^3^
Monoclinic, *P*2_1_/*n*	Mo *K*α radiation, λ = 0.71073 Å
Hall symbol: -P 2yn	Cell parameters from 4180 reflections
*a* = 8.351 (4) Å	θ = 1.6–27.8°
*b* = 12.461 (6) Å	µ = 0.12 mm^−^^1^
*c* = 11.912 (6) Å	*T* = 113 K
β = 100.315 (9)°	Prism, yellow
*V* = 1219.5 (11) Å^3^	0.24 × 0.22 × 0.20 mm
*Z* = 4	

### Data collection

**Table:**

Rigaku Saturn CCD area-detector diffractometer	2900 independent reflections
Radiation source: rotating anode	2459 reflections with *I* > 2σ(*I*)
Multilayer monochromator	*R*_int_ = 0.045
Detector resolution: 14.63 pixels mm^-1^	θ_max_ = 27.8°, θ_min_ = 2.4°
ω and φ scans	*h* = −10→10
Absorption correction: multi-scan *CrystalClear*	*k* = −16→16
*T*_min_ = 0.972, *T*_max_ = 0.977	*l* = −15→15
12466 measured reflections	

### Refinement

**Table:**

Refinement on *F*^2^	Primary atom site location: structure-invariant direct methods
Least-squares matrix: full	Secondary atom site location: difference Fourier map
*R*[*F*^2^ > 2σ(*F*^2^)] = 0.052	Hydrogen site location: inferred from neighbouring sites
*wR*(*F*^2^) = 0.131	H atoms treated by a mixture of independent and constrained refinement
*S* = 1.12	*w* = 1/[σ^2^(*F*_o_^2^) + (0.063*P*)^2^ + 0.1263*P*] where *P* = (*F*_o_^2^ + 2*F*_c_^2^)/3
2900 reflections	(Δ/σ)_max_ = 0.003
189 parameters	Δρ_max_ = 0.30 e Å^−^^3^
2 restraints	Δρ_min_ = −0.26 e Å^−^^3^

### Special details

**Table:**

Geometry. All e.s.d.'s (except the e.s.d. in the dihedral angle between two l.s. planes) are estimated using the full covariance matrix. The cell e.s.d.'s are taken into account individually in the estimation of e.s.d.'s in distances, angles and torsion angles; correlations between e.s.d.'s in cell parameters are only used when they are defined by crystal symmetry. An approximate (isotropic) treatment of cell e.s.d.'s is used for estimating e.s.d.'s involving l.s. planes.
Refinement. Refinement of *F*^2^ against ALL reflections. The weighted *R*-factor *wR* and goodness of fit *S* are based on *F*^2^, conventional *R*-factors *R* are based on *F*, with *F* set to zero for negative *F*^2^. The threshold expression of *F*^2^ > σ(*F*^2^) is used only for calculating *R*-factors(gt) *etc*. and is not relevant to the choice of reflections for refinement. *R*-factors based on *F*^2^ are statistically about twice as large as those based on *F*, and *R*- factors based on ALL data will be even larger.

### Fractional atomic coordinates and isotropic or equivalent isotropic displacement parameters (Å^2^)

**Table:**

	*x*	*y*	*z*	*U*_iso_*/*U*_eq_	
F1	0.29184 (14)	0.96861 (8)	−0.02312 (9)	0.0449 (3)	
O1	−0.04969 (14)	0.17411 (10)	0.34582 (10)	0.0322 (3)	
O2	0.01132 (14)	0.04318 (10)	0.24132 (10)	0.0340 (3)	
O3	0.19105 (13)	0.60131 (9)	0.05581 (9)	0.0277 (3)	
N1	0.01105 (16)	0.13960 (12)	0.26533 (12)	0.0265 (3)	
N2	0.26220 (16)	0.43681 (11)	−0.00884 (11)	0.0255 (3)	
N3	0.32644 (17)	0.58967 (11)	−0.09569 (11)	0.0258 (3)	
C1	0.20093 (18)	0.36633 (13)	0.06336 (13)	0.0236 (3)	
C2	0.19815 (19)	0.25683 (13)	0.03495 (13)	0.0252 (4)	
H2	0.2380	0.2346	−0.0312	0.030*	
C3	0.13910 (19)	0.18127 (13)	0.10071 (14)	0.0264 (4)	
H3	0.1371	0.1074	0.0808	0.032*	
C4	0.08209 (18)	0.21605 (13)	0.19783 (13)	0.0232 (3)	
C5	0.08776 (19)	0.32275 (13)	0.22924 (14)	0.0258 (4)	
H5	0.0505	0.3439	0.2967	0.031*	
C6	0.14717 (19)	0.39910 (13)	0.16335 (13)	0.0258 (4)	
H6	0.1517	0.4725	0.1852	0.031*	
C7	0.25437 (18)	0.54780 (13)	−0.01016 (13)	0.0234 (3)	
C8	0.35025 (18)	0.69898 (13)	−0.11978 (13)	0.0246 (4)	
C9	0.42473 (19)	0.72135 (14)	−0.21284 (14)	0.0268 (4)	
H9	0.4554	0.6642	−0.2573	0.032*	
C10	0.4543 (2)	0.82664 (14)	−0.24089 (14)	0.0297 (4)	
H10	0.5056	0.8406	−0.3044	0.036*	
C11	0.4106 (2)	0.91169 (15)	−0.17805 (15)	0.0327 (4)	
H11	0.4305	0.9840	−0.1969	0.039*	
C12	0.3367 (2)	0.88660 (13)	−0.08702 (15)	0.0307 (4)	
C13	0.30351 (19)	0.78355 (14)	−0.05540 (14)	0.0274 (4)	
H13	0.2509	0.7705	0.0077	0.033*	
H2A	0.314 (2)	0.4069 (14)	−0.0618 (13)	0.037 (5)*	
H3A	0.372 (2)	0.5429 (15)	−0.1377 (15)	0.056 (7)*	

### Atomic displacement parameters (Å^2^)

**Table:**

	*U*^11^	*U*^22^	*U*^33^	*U*^12^	*U*^13^	*U*^23^
F1	0.0653 (8)	0.0268 (6)	0.0451 (7)	−0.0026 (5)	0.0172 (6)	−0.0077 (5)
O1	0.0318 (6)	0.0391 (7)	0.0288 (7)	−0.0028 (5)	0.0136 (5)	0.0027 (5)
O2	0.0370 (7)	0.0269 (6)	0.0393 (7)	−0.0018 (5)	0.0096 (5)	0.0044 (5)
O3	0.0300 (6)	0.0275 (6)	0.0278 (6)	0.0027 (5)	0.0116 (5)	−0.0012 (5)
N1	0.0231 (7)	0.0283 (7)	0.0281 (7)	−0.0001 (6)	0.0044 (5)	0.0032 (6)
N2	0.0292 (7)	0.0241 (7)	0.0265 (7)	0.0010 (6)	0.0141 (6)	0.0009 (6)
N3	0.0303 (7)	0.0235 (7)	0.0265 (7)	0.0016 (6)	0.0130 (6)	0.0012 (6)
C1	0.0206 (7)	0.0276 (8)	0.0236 (8)	0.0013 (6)	0.0060 (6)	0.0015 (6)
C2	0.0259 (8)	0.0284 (8)	0.0228 (8)	0.0016 (6)	0.0084 (6)	−0.0001 (6)
C3	0.0257 (8)	0.0250 (8)	0.0287 (9)	0.0008 (6)	0.0056 (7)	−0.0005 (6)
C4	0.0213 (7)	0.0262 (8)	0.0228 (8)	−0.0009 (6)	0.0057 (6)	0.0044 (6)
C5	0.0259 (8)	0.0285 (9)	0.0250 (8)	0.0023 (6)	0.0097 (6)	−0.0002 (6)
C6	0.0281 (8)	0.0245 (8)	0.0267 (8)	0.0002 (6)	0.0099 (7)	−0.0007 (6)
C7	0.0205 (7)	0.0257 (8)	0.0239 (8)	0.0009 (6)	0.0042 (6)	0.0006 (6)
C8	0.0208 (7)	0.0254 (8)	0.0266 (8)	0.0015 (6)	0.0018 (6)	0.0022 (6)
C9	0.0250 (8)	0.0292 (9)	0.0263 (8)	0.0008 (6)	0.0051 (6)	0.0037 (7)
C10	0.0278 (8)	0.0320 (9)	0.0287 (9)	−0.0021 (7)	0.0032 (7)	0.0051 (7)
C11	0.0352 (9)	0.0275 (9)	0.0342 (9)	−0.0054 (7)	0.0027 (7)	0.0030 (7)
C12	0.0344 (9)	0.0250 (8)	0.0315 (9)	−0.0005 (7)	0.0029 (7)	−0.0035 (7)
C13	0.0286 (8)	0.0279 (8)	0.0257 (8)	−0.0018 (7)	0.0048 (7)	0.0011 (7)

### Geometric parameters (Å, º)

**Table:**

F1—C12	1.366 (2)	C3—H3	0.9500
O1—N1	1.2392 (18)	C4—C5	1.380 (2)
O2—N1	1.235 (2)	C5—C6	1.381 (2)
O3—C7	1.2209 (19)	C5—H5	0.9500
N1—C4	1.441 (2)	C6—H6	0.9500
N2—C7	1.384 (2)	C8—C9	1.393 (2)
N2—C1	1.389 (2)	C8—C13	1.399 (2)
N2—H2A	0.907 (9)	C9—C10	1.387 (2)
N3—C7	1.376 (2)	C9—H9	0.9500
N3—C8	1.413 (2)	C10—C11	1.384 (2)
N3—H3A	0.897 (9)	C10—H10	0.9500
C1—C2	1.405 (2)	C11—C12	1.376 (3)
C1—C6	1.407 (2)	C11—H11	0.9500
C2—C3	1.372 (2)	C12—C13	1.380 (2)
C2—H2	0.9500	C13—H13	0.9500
C3—C4	1.397 (2)		
			
O2—N1—O1	122.33 (14)	C5—C6—C1	118.87 (16)
O2—N1—C4	119.72 (14)	C5—C6—H6	120.6
O1—N1—C4	117.95 (14)	C1—C6—H6	120.6
C7—N2—C1	128.07 (14)	O3—C7—N3	124.54 (16)
C7—N2—H2A	115.5 (12)	O3—C7—N2	124.28 (15)
C1—N2—H2A	116.4 (12)	N3—C7—N2	111.18 (14)
C7—N3—C8	127.73 (14)	C9—C8—C13	119.58 (15)
C7—N3—H3A	116.9 (14)	C9—C8—N3	116.97 (15)
C8—N3—H3A	115.1 (14)	C13—C8—N3	123.46 (15)
N2—C1—C2	117.18 (14)	C10—C9—C8	120.35 (16)
N2—C1—C6	123.33 (15)	C10—C9—H9	119.8
C2—C1—C6	119.47 (15)	C8—C9—H9	119.8
C3—C2—C1	121.41 (15)	C11—C10—C9	121.22 (16)
C3—C2—H2	119.3	C11—C10—H10	119.4
C1—C2—H2	119.3	C9—C10—H10	119.4
C2—C3—C4	118.09 (15)	C12—C11—C10	116.83 (16)
C2—C3—H3	121.0	C12—C11—H11	121.6
C4—C3—H3	121.0	C10—C11—H11	121.6
C5—C4—C3	121.57 (15)	F1—C12—C11	118.40 (15)
C5—C4—N1	118.89 (14)	F1—C12—C13	117.07 (15)
C3—C4—N1	119.51 (15)	C11—C12—C13	124.52 (16)
C4—C5—C6	120.54 (15)	C12—C13—C8	117.49 (16)
C4—C5—H5	119.7	C12—C13—H13	121.3
C6—C5—H5	119.7	C8—C13—H13	121.3
			
C7—N2—C1—C2	168.79 (15)	C8—N3—C7—O3	4.1 (3)
C7—N2—C1—C6	−13.1 (2)	C8—N3—C7—N2	−176.20 (14)
N2—C1—C2—C3	−179.65 (14)	C1—N2—C7—O3	0.8 (3)
C6—C1—C2—C3	2.2 (2)	C1—N2—C7—N3	−178.89 (14)
C1—C2—C3—C4	−0.3 (2)	C7—N3—C8—C9	−179.05 (15)
C2—C3—C4—C5	−1.5 (2)	C7—N3—C8—C13	1.1 (3)
C2—C3—C4—N1	176.62 (13)	C13—C8—C9—C10	0.8 (2)
O2—N1—C4—C5	−176.89 (14)	N3—C8—C9—C10	−179.09 (14)
O1—N1—C4—C5	3.6 (2)	C8—C9—C10—C11	−0.2 (2)
O2—N1—C4—C3	4.9 (2)	C9—C10—C11—C12	0.0 (2)
O1—N1—C4—C3	−174.56 (13)	C10—C11—C12—F1	−179.98 (14)
C3—C4—C5—C6	1.5 (2)	C10—C11—C12—C13	−0.3 (3)
N1—C4—C5—C6	−176.63 (14)	F1—C12—C13—C8	−179.49 (14)
C4—C5—C6—C1	0.4 (2)	C11—C12—C13—C8	0.8 (3)
N2—C1—C6—C5	179.79 (14)	C9—C8—C13—C12	−1.0 (2)
C2—C1—C6—C5	−2.1 (2)	N3—C8—C13—C12	178.82 (15)

### Hydrogen-bond geometry (Å, º)

**Table:**

*D*—H···*A*	*D*—H	H···*A*	*D*···*A*	*D*—H···*A*
N2—H2*A*···O1^i^	0.91 (1)	1.99 (1)	2.890 (2)	170 (2)
N3—H3*A*···O2^i^	0.90 (1)	2.28 (1)	3.157 (2)	168 (2)

Symmetry code: (i) *x*+1/2, −*y*+1/2, *z*−1/2.
